# Death of Dr. H. T. Babcock

**Published:** 1879-01

**Authors:** 


### Death of Dr. Heman P. Babcock of Oakland, California.

We have only space in this issue to chronicle the death of Dr. Heman P. Babcock of Oakland, Cal., which occurred in this city at the residence of Prof. Thomas F. Rochester. Dr. B. had been in declining health for the past seven months, and took the journey from Oakland for the purpose of consulting Prof. Rochester, his former Preceptor and friend, in whom he had the utmost confidence, and at whose hands he received every attention that love and skill could bestow, but all to no avail; he quietly passed away on the morning of Friday, Dec. 27th. The funeral was attended on Monday at 11 o'clock from the residence of Prof. Rochester.

We shall have more to say of Dr. B. hereafter.

